# Consumption of nutritive supplements during physical activities in Romania: A qualitative study

**DOI:** 10.1002/fsn3.2990

**Published:** 2022-07-27

**Authors:** Romeo Victor Ionescu, Valentin Marian Antohi, Monica Laura Zlati, Mihaela Teodora Iconomescu, Alexandru Nechifor, Silvius Stanciu

**Affiliations:** ^1^ Department of Administrative Sciences and Regional Studies Dunarea de Jos University of Galati Galati Romania; ^2^ Department of Business Administration Dunarea de Jos University of Galati Galati Romania; ^3^ Department of Finance, Accounting and Economic Theory Transylvania University of Brasov Brasov Romania; ^4^ Department of Accounting, Audit and Finance Stefan cel Mare University of Suceava Suceava Romania; ^5^ Department of Sports Games and Physical Education Dunarea de Jos University of Galati Galati Romania; ^6^ Department of Medical Clinical Dunarea de Jos University of Galati Romania; ^7^ Department of Food Science, Food Engineering, Biotechnology and Aquaculture Dunarea de Jos University of Galati Romania

**Keywords:** econometric model, efficiency, nutrients, nutritional supplements consumption, sports

## Abstract

The consumption of nutritional supplements by the population that practices physical activities in a regular, organized manner represents a form of scientific interest for professionals interested in analyzing the somatic and biological changes that occur under the influence of the stimuli provided by nutritional supplements. In this context, based on a new statistical model proposed by the authors, we aimed at assessing nutritional supplement consumption efficiency and the effects felt by the consumers in relation to both the achievement of their health goals and the destructuring of the biological processes following the consumption of these supplements. The new model, created by the authors, can be applied to the population of any other state in the world. The methods used in this article are analytical and prospective, and they are based on a qualitative questionnaire applied to 310 Romanian people who practice sports regularly; the sample group being considered representative for the entire population of Romania by the Cochran W.G. test. The results of the study are useful to both the specialists and the people who take nutritional supplements, and they help in improving the perception regarding the efficiency of these products on various categories, as will be seen from the data provided in this study. Moreover, the results of this analysis are also interesting for the providers of products such as pharmacies and authorized distributors, which can scientifically quantify their supply in the market.

## Graphical abstract

The first key finding presented in the paper is assessing nutritional supplement consumption efficiency and the effects felt by the consumers in relation to the achievement of their health goals and to the destructuring of the biological processes, following the consumption of these supplements. The second key finding covers the destructuring of biological processes, which reveals the acceptance regarding the effectiveness of nutritional supplement use. Finally, the article points out the differences between the male and female population’s behaviour.

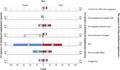

## 1 INTRODUCTION

The consumption of dietary supplements is widespread among both professional athletes and sports enthusiasts.

Dietary supplements are used for nutrient deficiency or very strict diets. Their use is often considered necessary in professional or amateur sports to improve physical fitness. Buying dietary supplements by the people who want to use them is very easy as they are sold without a prescription in pharmacies, gyms, and on the Internet. Amateur or professional athletes often take supplements without thinking about the side effects, mainly because they are substances that improve physical fitness (Antonio, 2019; Antonioni et al., 2019). This is why it is widely used among athletes at all levels, reflecting the prevalence of their use in society. Most athletes use supplements for the following reasons: muscle building, mineral loss, to be able to increase exercise intensity, recovery after exercising, and optimize weight and body composition (Antonio, 2019; Đorđević‐Nikić & Đorđević, 2006).

In some countries, the production and sale of dietary supplements is not so strict, with illegal substances being found in dietary supplements. Athletes buy supplements on the Internet without considering the possibility of doping or endangering their health (Đorđević‐Nikić & Đorđević, 2006; Savino et al., 2019).

That is why there is a need for continuous education on the principles of the use of dietary supplements by athletes in order to develop good dietary habits and maintain their health.

Most specialists have researched the topic of the use of supplements by professional athletes, but there is a gap in the research regarding their use by amateur athletes (Blennerhassett et al., 2019; Đorđević‐Nikić & Đorđević, 2006; Savino et al., 2019).

The study of nutrients is a perpetually innovative tool for the industry, arousing the interest of the vast majority of specialists both by means of quantitative methods, used to improve the effects of consumption on the population who practices physical activities by increasing body performance, and by means of qualitative methods, used to improve nutrient intake by using quality amino acids, creatinine, or fat burning supplements (Chacón‐Cuberos et al., 2019; Jovanov et al., 2019; Prowse et al., 2018). Another parallel, drawn by nutritionists and diabetologists, is the combination, in a balanced diet, of fats, trace elements, quality proteins, and omega‐3 fatty acids (Dunford & Doyle, 2018; Espinosa et al., 2018; Guest et al., 2019). Thus, the pharmaceutical industry has marketed a large number of nutritional supplements designed to intensify the effects of sustained effort. The most popular nutritional product ranges sold in pharmacies and in online stores cover the following product categories: amino acids, antioxidants, protein bars to speed up the weight loss process, carbohydrates, digestive enzymes, creatinine, drinkable nutritional products, glutamine‐based products, products for immunity, neurotransmitters, products based on nitric oxides, products especially designed for athletes, products especially designed for women, probiotics, protein powders, vitamins, nutrient dispensers, etc. (El Khoury et al., 2019; Maughan et al., 2018; Šterlinko Grm et al., 2012).

The nutrients are a topic of real interest in multidisciplinary research, both from the perspective of their effects on the human metabolism and from a purely economic perspective on production, marketing, and economic efficiency in a sustainable context (Caraballo et al., 2020; Close et al., 2016; Lee et al., 2019; Manore et al., 2017).

The analysis of the literature highlights the continuous concern of researchers regarding the relationship between nutrition–physical activities–metabolism. On the other hand, the differences in approach do not cover the full range of issues in this nutrition–physical activities–metabolism relationship (Mountjoy et al., 2018; Paoli, 2019; Thorning et al., 2017; Ward et al., 2019).

Due to the extremely diversified product ranges, the existence of a very large number of suppliers and the large and expanding global demand, our scientific approach is useful to both specialists and consumers. The general goal of this research is to quantify the efficiency of nutritional supplement consumption in the case of the population who regularly practices at least one physical activity and to analyze the positive and negative effects induced by the consumption of such supplements.

The following specific objectives of the research may be defined:

*O1*: To quantify the efficiency of nutritional supplement consumption in the case of the population who practices a sport.
*O2*: To quantify the effects felt by the consumers in relation to the achievement of their health goals.
*O3*: To quantify the effects felt by the consumers in relation to the destructuring of the biological processes following the consumption of these supplements.

These objectives will contribute to the improvement of the nutritional picture by correlating the biological mechanisms with the psychological and volitional ones and by selecting the mechanisms used by consumers in establishing the mix of usable nutrients in relation to the proposed goal.

In this context, the new model (presented in this scientific approach) is intended to clarify the methodological and practical aspects of the relationship between sport and diet – performance from both a cognitive and a perceptual point of view.

## 2 METHODS

### 2.1 Study design

**FIGURE 1:**
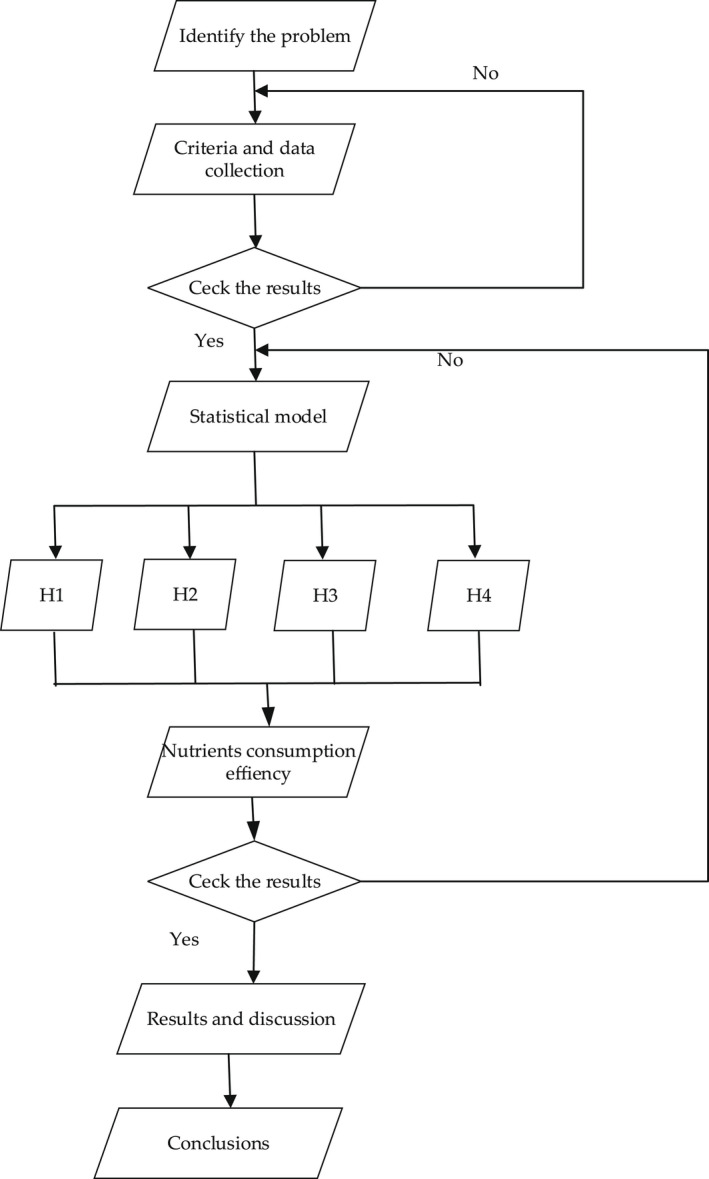
Research algorithm

This methodology is structured according to a certain pattern of the research and is in line with the set objectives (nutritional supplement consumption efficiency and the effects felt by consumers in relation to both achieving health objectives and destructuring of biological processes following the consumption of these supplements) and the obtained results (see Figure 1).

This study was approved by the Ethics Committee of the Faculty of Physical Education and Sport, Dunarea de Jos University of Galati, and all the procedures were conducted in accordance with the Declaration of Helsinki and participants gave informed consent before participating in the study.

There were certain inclusion criteria that were taken into account. Some compulsory conditions were: to practice a physical activity, to reside in Romania, and to graduate a tertiary education. Sedentary people were excluded from the sample group.

### 2.2 Participant characteristics

In order to determine the causal relations between the consumption of nutritional supplements and the physical activities, the interview was based on a questionnaire, used for 310 respondents. Quantitative data were collected between December 2019 and May 2020, the participants being from the southeast region of Romania.

According to the test designed by Cohran W.G., the sample group is representative for the population of Romania (19 million inhabitants) in the case of 289 respondents, at an estimated probabilistic incidence of 25% and a sample accuracy rate of −0.05, with a confidence interval of 95% and an assigned constant *c* of 1.96.

### 2.3 Measurements

The structure of the questionnaire, which was in line with the objectives of the study, analyzed the physical activity of the subjects (gym attendance, the type of sport practiced, the motivation for practicing the respective sport), the acceptance of nutritional supplements in the diet, and specific data on the consumption of nutritional supplements (the goal of using supplements, the perceived utility of nutritional supplements, their efficiency, the frequency of consumption, types of nutritional supplements).

The questionnaire was followed by the analysis of the adverse effects caused by the consumption of nutritional supplements as well as the analysis of consumption options (motivation for selecting a certain nutritional supplement brand, logistics chains accessed in the supply process, the nutritional facts written on the label). The structural data collected from the respondents provide information on: age, sex, height, weight, information on body mass index (BMI) and nutritional status, marital status, occupation, education level, and the respondents' income.

The BMI for the assessment of ideal weight is based on height and weight and is a standard recommended by the World Health Organization. On the other hand, weight could be linked to sex sometimes.

The BMI is a surrogate marker of adiposity calculated as weight (kg)/height^2^ (m^2^). The BMI categories for defining overweight vary by age and gender in infants, children, and adolescents (World Health Organization, 2022).

The sports practiced by the respondents fall into three types of effort: aerobic, anaerobic, and mixed.

The level of representativeness mentioned earlier is reached, the total number of respondents being 310 people. Data analysis in the questionnaire reveals that there is a relatively symmetrical distribution between consumers and nonconsumers of nutritional supplements.

### 2.4 Statistical analysis

The analysis is based on the following *hypotheses*:

*H1*: The distributions of *age*, *height*, and *weight* are the same across the categories of *acceptance* of nutritional supplements (sports), where *age* – annual distribution of the sample population; *height* – height distribution in cm of the sample population; *weight* – weight distribution in kg of the sample population; *acceptance* – logical variable (type *yes* and *no*) for the study of the acceptance of nutrient consumption within the analyzed sample.
*H2*: The distribution of *weight* is the same across categories of *motivation* for physical activity, *type* of practiced physical activity, and the gym attendance, where *motivation* represents the motivation for sports evaluated on the following qualitative levels: aesthetic motivation, the pleasure of practicing physical activity, medical motivation, at least two of the above reasons; type of practiced physical activity – the sample analysis for fitness/strength/cardio, football, volleyball, aerobics (Tae‐Bo, Zumba, step aerobics, Kangoo jumps), basketball, pilates, spinning, other sports; at least two sports; frequency at the gym – frequently, more than four times a week, once a week, more than four times a month, once a month.
*H3*: The distribution of *age* is the same across categories: frequency of nutritional supplement consumption, the purpose of using nutritional supplements, and the usefulness of nutritional supplements, where frequency of nutritional supplement consumption (daily or weekly) structured according to the intensity of the training program; do not exceed the recommended daily dose; the purpose of using nutritional supplements: recovery after effort, development of muscle mass, decreasing the percentage of fat, weight loss, increased muscle strength, at least two of the above purposes; the usefulness of nutritional supplements: the introduction of nutrients that the consumers cannot assimilate through daily diet, combined with diet, helps consumers increase their muscle mass; they give consumers more stamina; combined with diet it helps consumers lose weight; at least two of the above options.
*H4*: The distribution of *age* is the same across categories of motivation for selecting the nutritional supplement brand, where motivation for selecting covers: recommendation of a specialist; loyalty to a certain brand; accessibility in point of price.

The model we propose, based on cumulative regression equations, analyses the acceptance of nutrient consumption, by means of the Wald–Wolfowitz independent statistical tests. Depending on the respondent’s age criterion (dependent variable), the linear model was designed based on the least squares method for the following regression variables: frequency at the gym (FG); the type of practiced physical activity (PS–PPA); motivation for physical activity (MS–MPA); acceptance of nutritional supplements (sports) (AN); the purpose of using nutritional supplements (PRP); the usefulness of nutritional supplements (USN); effectiveness of nutritional supplements (EFF); and frequency of nutritional supplement consumption (FRQ).

By means of statistical modeling, a statistically significant score function was obtained on the whole level of regressors below 50%, which leads to the conclusion that there are, within the sample structured on age levels, heterogeneous options for at least 50% of the modeled regressors. The statistical data are presented in Table 1.

**TABLE 1:** Regression statistical model for the age‐dependent variable indicator in relation to defined regressors

Model summary^b^						
Model	R	*R* ^2^	Adjusted *R* ^2^	Std. error of the estimate	Change statistics	
					*R* ^2^ change	*F* change
1	0.183^a^	0.034	0.006	7.923	0.034	1.238

Model summary^b^				
Model	Change statistics			
	*df*1	*df*2	Sig. *F* change	
1	8	285	0.277	2.061

^a^ Predictors: (Constant), frequency of nutritional supplement consumption, the type of practiced physical activity, motivation for physical activity, frequency at the gym, the purpose of using nutritional supplements, the usefulness of nutritional supplements, effectiveness of the use of nutritional supplements, acceptance of nutritional supplements (sports).

^b^ Normality test – Null hypothesis: the error is normally distributed. Statistic test: Hi^2^ = 30.09 with *p* = 2.92443e‐007. Dependent variable: Age.

From the frequency series distribution, by using the normality test of the residues, it is found that in terms of nutrient consumption acceptance in relation to the physical activities' frequency, the motivation and the goal considered by those practicing physical activity, the trend is given by the young people, up to 30 years of age. They determine the consumer behavior in 98.6%, which places the consumption of nutrients on the segment of volute psychology related less to the quality of the consumed products and more to the goal pursued by the consumption action (e.g., increasing self‐esteem).

Thus, the possibility of a double model analysis appears on several segments of the structure, that is, the respondents' age, their physical and biological characteristics, the occupational characteristics, or the income level of the respondent population. Due to the writing standards of an article and its relatively limited length, the authors limited themselves to the analysis based on the segmentation criterion of the respondents' age, because it represents the most significant variable in relation to the research objective. In order to assess the volitional and psychological level in opposition to the analytical and qualitative level, we devised two statistical models, based on the least squares method, regarding the sample group structure segmented by age. On one hand, the trend equations will be calculated based on the psychological and volitional components, and on the other hand, they will be calculated by taking into account the analytical and qualitative components, as follows:

Model 1 psychological–volitional: presents the following equation.

(1) ^Age=+1.00×FG+2.24×PPA+4.70×MPA+0.630×PRP

$$\left(0.347\right)\left(0.273\right)\left(0.458\right)\left(0.320\right)$$

n=310,R−squared=0.906

$$\left(\text{standard errors in parentheses}\right)$$

where age is the dependent variable according to which the sample is segmented (age in years); FG – frequency at the gym; PPA – the type of practiced physical activity; MPA – motivation for practicing a physical activity; PRP – the purpose of using nutritional supplements; *n* – number of people in the questionnaire.

There is a high statistical significance of the model (over 90%) and an intensity of correlation proven by level 1 statistical tests of regressors on motivational factors (FG, MPA, PPA). Regarding the purpose of using supplements, the level of correlative intensity is lower (the value is 3).

The data presented prove the validity of the model for which the null hypothesis confers results regarding the normally distributed error and the absence of heteroscedasticity, with a statistical significance of over 90%.

Model 2 analytical–qualitative: presents the following equation:

(2) ^Age=−4.02×AN+4.22×USN+5.27×EFF+4.63×FRQ

$$\;\left(1.60\right)\left(0.976\right)\left(1.32\right)\left(1.51\right)$$

n=310,R−squared=0.696

$$\left(\text{standard errors in parentheses}\right)$$

where age is the dependent variable according to which the sample group is segmented (age in years); AN – acceptance of nutritional supplement; USN – the usefulness of nutritional supplements; EFF – effectiveness of nutritional supplements; FRQ – consumption frequency of nutritional supplements; *n* – number of people in the questionnaire.

There is a statistical significance of the model below 70% and a level 1 correlation intensity, proven by statistical tests of the regressors regarding the analytical–qualitative factors (USN, EFF, FRQ). Regarding the acceptance of nutrient consumption, the level of correlative intensity is 2 (lower).

From the data presented above, it results that the model is valid, homogeneous, with a normally distributed error in the null hypothesis, and the heteroscedasticity is absent. The analytical–qualitative model has a lower statistical significance than the psychological–volitional model. The modeled data represented by statistical distribution plot diagrams, with the help of the SPSS 25 program, reveal the preliminary conclusions, obtained after development of the two types of models.

## 3 RESULTS AND DISCUSSION

As a result of the methodological study, the authors found that the consumption of nutritional supplements in physical activities has a more accentuated effect on the psychological component of the people practicing a sport. However, the qualitative elements were also analyzed related to this consumption, such as frequency of consumption in relation to physical activity and recommended daily dose, the selection from the varied offer of a personalized consumption configuration according to the type of physical activity carried out and the proposed purpose, and the analysis of the technical information on the leaflet, the recommendations made by specialists.

Taking into account the frequency scale (1 – once a month; 2 – once a week; 3 – more than four times a month; 4 – more than four times a week; 5 – frequently) for the FG – frequency at the gym indicator: the frequency distribution for the dependent variable in relation to the gym attendance indicator illustrates that the concentration is achieved on the fourth and fifth levels for the average age range 25–30 years. As a result, the polarization is performed in the upper part of the age range toward the lower frequency limit and in the opposite direction with maximum polarization in the lower age limit area of 20–30 years.

Similarly, for the PPA indicator, according to the options presented in the questionnaire, the intensity scale was set by the authors depending on the type of the physical activity, as follows: 1 – spinning, pilates; 2 – football; 3 – volleyball, basketball; 4 – aerobics (Tae‐Bo, Zumba, step aerobics, Kangoo jumps); 5 – fitness/strength/cardio. There is a polarization of the frequency range of the age‐dependent variable on the midrange area of physical activity which presupposes sustained effort (fourth and fifth levels of intensity) with reduced intensity of physical activities on the upper age levels and increase in intensity in the lower age limit of respondents.

The motivational factors are analyzed on an intensity scale (1 – other; 2 – aesthetic motivation; 3 – the pleasure of practicing physical activity; 4 – medical motivation; 5 – at least two of the above reasons) that places the pleasure of practicing a physical activity in the midrange area and the medical motivations or the motivational multiples at the upper limit of the motivational intensity interval. From a motivational point of view, the data reflect the fact that the model is well structured, centered on the midrange area from the point of view of the structure of the dependent variable, at the intersection with the midrange area for the motivational regressor.

Level 5 intensification occurs at the lower limit of the age range, while at the upper limit, the values of motivational input tend to decrease, which confirms that the motivational stimulus is especially specific to the young population.

**FIGURE 2:**
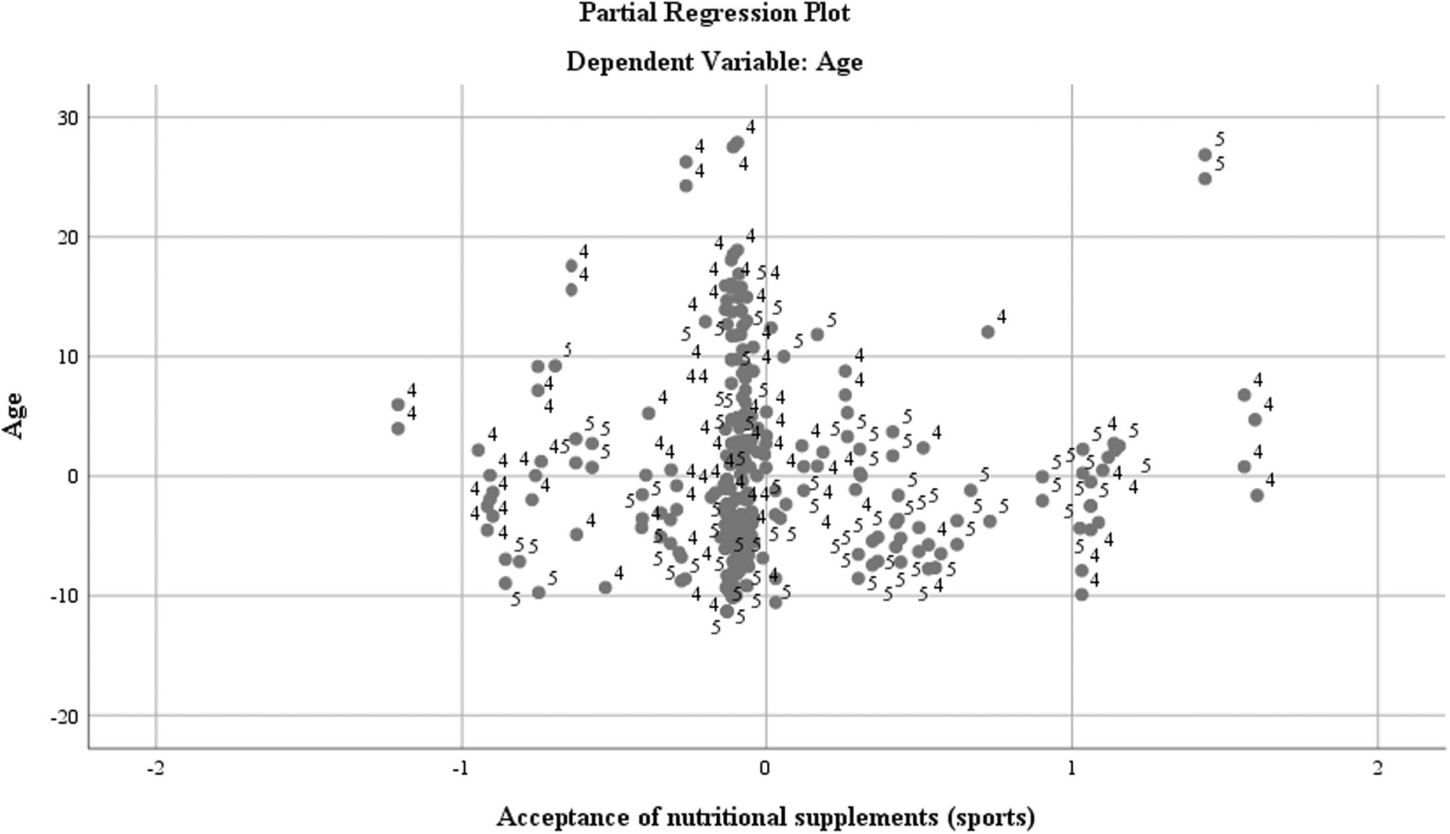
Regression analysis using the dependent variable age and the acceptance of nutritional supplements

The acceptance of nutritional supplements seen as a logical variable in the design of the questionnaire (see Figure 1) reflects (for respondent users) a distribution structured in the lower age limit (see Figure 2).

The same polarized structure in the midrange area is found in the case of the regressor model, namely PRP. According to the design of the questionnaire, the intensity structure was established by the authors as follows: 1 – decreasing the percentage of fat; 2 – development of muscle mass; 3 – weight loss; 4 – increased muscle strength; 5 – recovery after effort or at least two of the above purposes (see the Appendix A).

There is a frequency concentration at the intersection of the mid values of the age range regarding the use of nutritional supplements. On the other hand, in the quartile inflection points of the frequency range, regarding the purpose of nutrient use, clusters may be identified on the lower age segment, while on the upper age segment the concentration is achieved around the middle of the frequency range for this purpose.

The intensity scale for the consumption utility indicator (USN) was devised by the authors: 1 – no; 2 – more energy; 3 – lose weight; 4 – muscle mass; 5 – nutrients that I cannot assimilate through daily diet.

**FIGURE 3:**
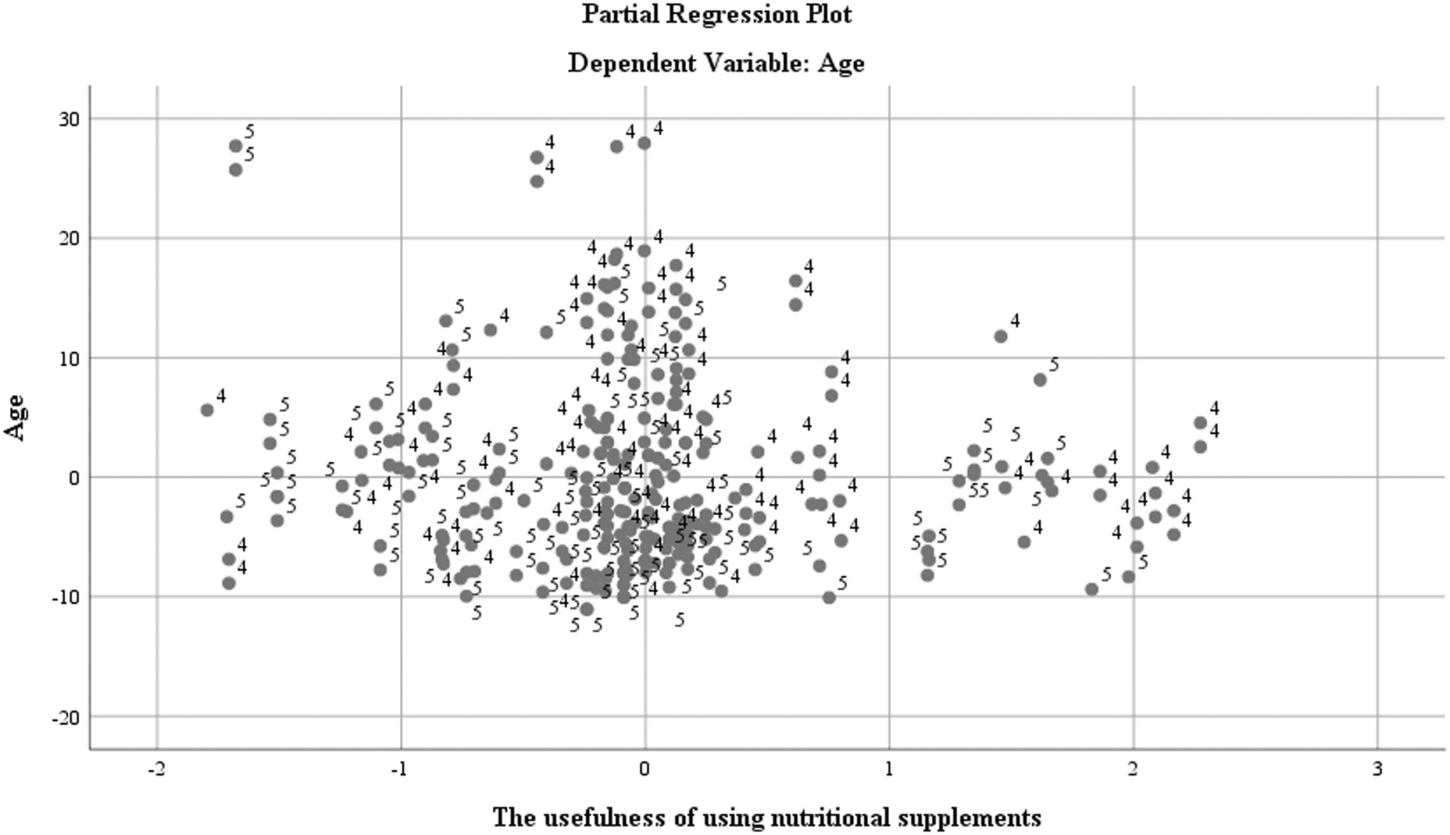
Regression analysis using the dependent variable age and the usefulness of nutritional supplements

By using the correlative distribution between the dependent variable and the analyzed regressor, it results that there is a semiconcentrated distribution on the midrange age and utility interval according to the data presented in Figure 3.

Regarding the efficiency of using nutritional supplements, this corresponds to the upper end of the finality desired by the users (acceleration of metabolic processes) on regressively structured levels, from the threshold of 30% (maximum range) to the minimum threshold or absence of nutrient consumption effects.

The polarization is clear, situated on the right side of the efficiency frequency range, with significant distribution in the lower age segment. In the same way, one may notice that the behavior of the sample group structured according to the average age in terms of supplement consumption frequency. The correlative values are stronger in this case, the structure being favorable to the lower average age of the sample group. The scale of representation is structured according to the daily, weekly, or recommended level of consumption.

The phenomenon of accepting nutritional supplements has distinct characteristics depending on the consumer's sex, nutritional supplements being more intensely used by the male population, who practice physical activities of different types frequently and very frequently (daily or more than four times a week). Among the female population, there is a medium to low acceptance, the peak of acceptance being for women who practice a physical activity more than four times a month (weekly) (Caraballo et al., 2020; Nakhostin‐Roohi & Asadi, 2018).

In the case of women who practice physical activity frequently (daily), the level of acceptance is 50%, and among women who practice very rarely (once a month), there was a quasi‐unacceptability, this aspect being found among the male population.

From the type of practiced physical activity point of view, the highest level of acceptance is manifested by the segment of the male population who practices high‐intensity physical activities (fitness/bodybuilding/cardio training) and football. Among the female population, with the exception of basketball, volleyball, and football, the other women who practice a physical activity do not agree with the consumption of nutritional supplements (Caraballo et al., 2020; Nakhostin‐Roohi & Asadi, 2018). Women practicing aerobic activities represent the highest peak of net unacceptability, since they consider these activities as a motivation in themselves and they do not seem to need nutritional supplements.

In terms of motivational factors, the histogram distribution by sex structure shows an inversely proportional acceptance in favor of the male population, who practices a physical activity for the pleasure the training. A high net acceptance among the male population is also found in the case of practicing physical activity for medical reasons as well as in the case of strongly causal motivations (aesthetic, medical, and social motivations). As we pointed out earlier, the behavior of the female population is inversely proportional, the peak of net acceptance manifesting itself on the segment the pleasure of doing physical activity, but this peak is a negative one, the unacceptance on this segment exceeding acceptance.

As far as the use of nutritional supplements is concerned, one may notice that the population accepts the supplements used for muscle mass development and for post workout recovery or weight loss (Espinosa et al., Espinosa et al., 2018; Hartmann & Siegrist, 2016; Wardenaar et al., 2016). The distribution is favorable for the female segment regarding supplements intended for post workout recovery and for the male segment regarding muscle mass development supplements. There is a homogeneous distribution in the case of using supplements for weight loss. Concerning the use of supplements in order to decrease the percentage of fat, the acceptance is manifested only among women and the motivational process is a weak one, because this variable is poorly represented in the consumer choices.

**FIGURE 4:**
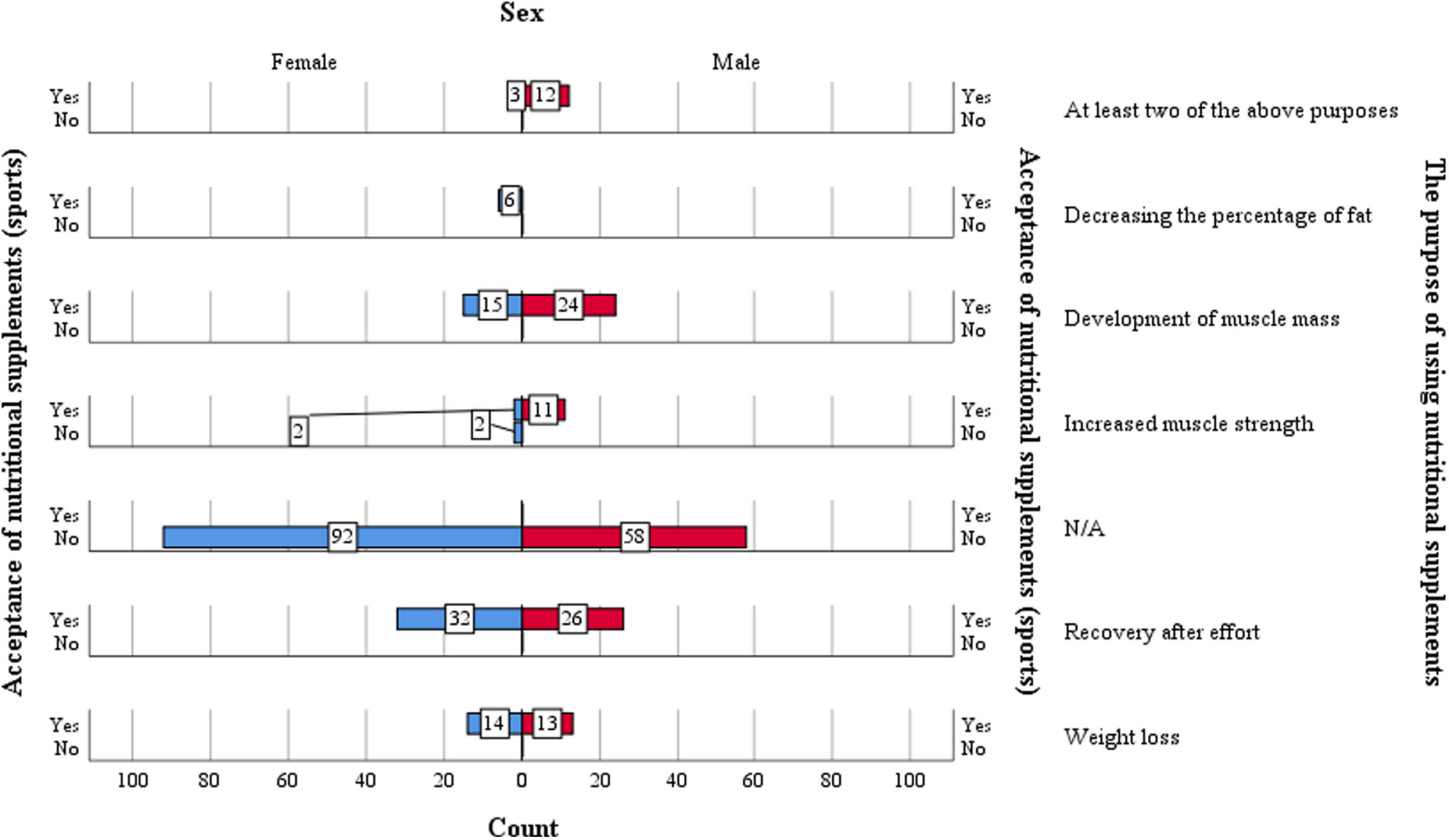
Acceptance of nutritional supplements according to sex/the purpose of using nutritional supplements

Furthermore, the ratio of women who show unacceptability toward the use of nutritional supplements as compared to that of men is 2:1 (see Figure 4).

The nutritional supplement usage efficiency is highlighted by the questionnaire‐based study on the women segment in association with the increase in stamina obtained after using supplements. On the other hand, in the case of men, the representativeness of acceptance is higher for the efficiency of increasing muscle mass. Also, there are homogeneous distributions in the case of weight loss efficiency and the assimilation of nutritional supplements, complementary to the daily diet.

The effectiveness of nutrient consumption in relation to the acceleration of metabolic processes is manifested on the lower level (acceleration of metabolic processes by 10%). There is a distribution in favor of the female population for which this efficiency motivates the use of supplements. On the next level (acceleration of metabolic processes by 20%), there is homogeneous acceptance among the male and female population. On the upper level (acceleration of metabolic processes by more than 30%), the male segment is much better represented and motivated by this efficiency obtained after the use of supplements (Espinosa et al., Espinosa et al., 2018), the distribution ratio being 3:1 in favor of the male population.

The consumption frequency of nutritional supplements demonstrates, for the moderate frequency levels (limiting the consumption to the amount of the recommended daily dose), a favorable representation of 2:1 for the male population. In the case of structured consumption, according to the intensity level of training, the female segment is better represented. The most frequent nutrient consumption (weekly/daily) presents a layering in favor of the male population (Caraballo et al., 2020; Espinosa et al., Espinosa et al., 2018; Hartmann & Siegrist, 2016), with a representation ratio of 1.5:1, with the mention that these frequency series are the least represented on the consumption scale (see Figure 2).

In terms of side effects, the majority of the female and male population who showed acceptance for consumption are positioned on the segment of supplements that do not generate side effects (Caraballo et al., 2020). There is a very small percentage (<3%) of the population that declares adverse effects due to nutritional supplement consumption.

The sort‐type–dimensional structure of nutrient consumption reflects a consumption distribution in favor of the female segment (3:1 representation) regarding supplements which are also used by athletes and of 1.5:1 for the consumption of supplements for fat burning. Male consumer preferences for protein and creatinine consumption are better represented (4:1 degree of representation). The protein consumption for increasing muscle mass as well as the simultaneous consumption of several types of nutrients is specific to men (2:1 representation ratio) (Nakhostin‐Roohi & Asadi, 2018).

Brand affiliation is specific to men (2:1 representation ratio). Moreover, price accessibility has a correlation of the same meaning in favor of the male population (3:1 representation ratio), both being motivations for acceptance. The specialist (Guest et al., 2019; Silva & Paiva, 2016) recommendation has a relatively homogeneous distribution slightly in favor of the female population.

The logistical chains prove that men accept nutritional supplements if they are distributed by means of authorized distributors (1.5:1 representation ratio). Another supply chain, that is, online commerce, is more specific to men (representation ratio 1.3:1), while at the opposite pole, the purchase of supplements from pharmacies proves to be a specific action for the female population.

**FIGURE 5:**
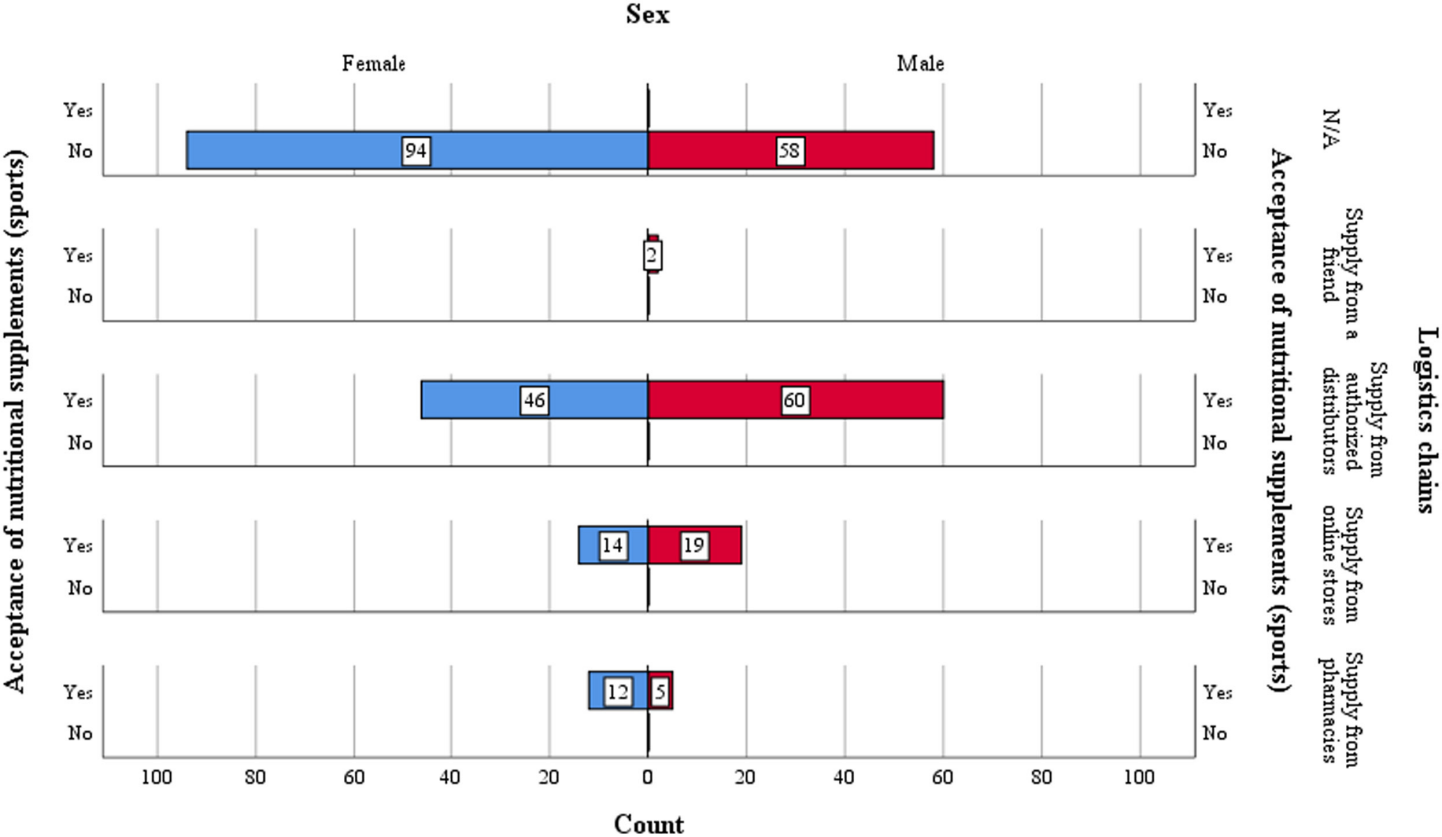
Acceptance of nutritional supplements according to sex/logistical chains

The study of the analyzed sample group shows that, in a very small percentage, only the male population uses acquaintances or friends to purchase nutritional supplements. The observations demonstrate that acceptance takes place in the context of the existence of reliable logistical chains, able to provide professional feedback, which is a qualitative attribute of the current nutritional supplement consumption, complementary to practicing a physical activity (see Figure 5).

When it comes to being familiar with the nutritional facts of a particular supplement, the vast majority of people participating in the study, both female and male, who use nutritional supplements, read the information about nutritional facts. There is a small redundant percentage of consumers who are not familiar with the nutritional facts, the level of representation being 4:1 in favor of the male population.

When taking into account the BMI, most people who accepted the use of nutritional supplements are those with a low BMI and good health. The positive net acceptance curve for women is maximized on the BMI of 25–29.9. The same table threshold is reached in the case of the male population, but the peak is much better defined, the ratio between acceptance and nonacceptance being 2:1 in this case (in the case of the female population it is 1:1). On the lower segment (18.5–24.9), the female acceptance is lower than unacceptance (representation index 0.75), while the male acceptance has a representation index of 1:1.

As far as weight is concerned, the structure of people practicing a physical activity and, at the same time, using nutritional supplements, is for the categories of professional athletes on the lower range of normal weight and for regular people practicing physical activity on the medium range with a tendency to obesity (two peaks) (Garthe & Maughan, 2018; Ward et al., 2019). For people who practice a physical activity occasionally, the structure is heterogeneous, with three inflection points.

From an occupational point of view, the greatest acceptance among women is manifested by women who practice physical activity, followed by women in professional training (students) and professional sportswomen. Instead, among men, the maximum acceptance level is manifested by athletes and students, followed by the military personnel.

The acceptance of nutritional supplements increases together with an increase in the consumers' educational level, the male student population having a representativeness of 2:1 compared to the population of the opposite sex. The acceptance increases together with an increase in income. In the case of men, the acceptance has a representation ratio of 4:1 on the maximum level.

The study shows that the biological mechanisms are correlated with the selection mechanisms used by consumers in determining the mix of nutritional supplements in relation to the proposed purpose. Through this study, all the research objectives were achieved, the working hypotheses were demonstrated, the main conclusion being that nutritional supplement consumption is mainly influenced by the psychological–volitional model (Sirico et al., 2018; Tabacchi et al., 2015) and alternatively by the analytical–qualitative model for establishing the combination of supplements used and their acceptance in the daily diet.

## 4 CONCLUSIONS

By means of the conducted research, the authors aimed at determining the acceptance of nutritional supplements by people practicing a physical activity. Accordingly, we devised a questionnaire whose structure was defined in the Methods, the representativeness of this questionnaire being demonstrated for the people who practice a physical activity in Romania, with the mention that the results of the study can be extended to other countries through procedural parallelism.

According to the statistical analysis, three hypotheses were put forward for testing acceptance, quantifying the efficiency of consumption and quantifying the effects felt by consumers.

Thus, the acceptance was tested with the help of independent statistical test Wald–Wolfowitz for all the main structural indicators (age, height, weight), the psychological and analytical parameters being modeled only through the age indicator, which was considered representative.

The *first working hypothesis* reflects the normally distributed acceptance in the null hypothesis, according to all the three structural indicators (age, height, weight).

The sigma index of statistical significance for the Wald–Wolfowitz independence test generated the maximum value 1, which indicates 100% statistical significance for the studied phenomenon and retains the null hypothesis. Thus, the working hypothesis H1 is validated as it was formulated, namely: the distributions of *age*, *height*, and weight are the same across categories of *acceptance* of nutritional supplements (sports).

The *second working hypothesis* was defined for testing the acceptance in relation to the weight of the respondents (BMI), finding sigma values of average statistical representativeness (up to 60%), maintaining the null hypothesis by the Jonckheere–Terpstra significance tests, test for ordered alternatives. Although the statistical significance does not support the uniformity of the motivational distribution on BMI levels, it can be changed between coastline or inner countries or as a result of weather range. The hypothesis is still valid, statistically proven by keeping the null hypothesis, namely: the distribution of *weight* is the same across categories of motivation for physical activity, type of practiced physical activity, and gym attendance.

The *third hypothesis* analyses the distribution of the analytical–qualitative factors in relation to the age structure indicator and is demonstrated by calculating the Kruskal–Wallis and Jonckheere–Terpstra indices, tests that retained the null hypothesis for all analyzed factors. The sigma value is included on the average statistical significance level of up to 80%. The authors showed in the methods section that the psychological–volitional model precedes the analytical–qualitative model in terms of statistical significance, the validity of the hypothesis being still demonstrated: the distribution of age is the same across categories of frequency of consumption of nutritional supplements, the purpose of using nutritional supplements, and the usefulness of nutritional supplements.

Brand affiliation is analyzed by the Jonckheere–Terpstra test, the level of statistical significance being over 80%, the null hypothesis being kept and the study hypothesis validated: the distribution of age is the same across categories of motivation for selecting the nutritional supplement brand.

With regard to the research objectives, the analysis showed that there is an average acceptance of nutritional supplement consumption, and the level of consumption efficiency is higher, in the sense that the increased energy levels directly influence consumption. The structure is favorable to the female population, in a ratio of 1.5:1. The *first objective* of the study is thus demonstrated, namely: quantifying the efficiency of nutritional supplement consumption in the case of the population that practices a physical activity and its directly proportional relationship with acceptance.

Taking into account the effects felt by the consumer, the test results clearly indicate the positioning of male and female consumers (over 90%) on the segment of products that do not generate adverse effects. In this way, the *second objective* is demonstrated: quantifying the effects felt by consumers in relation to achieving health goals and their directly dependent relationship with acceptance.

According to the study, the destructuring of biological processes, that is, in which metabolic processes suffer an acceleration of 10%, reveals the acceptance regarding the effectiveness of nutritional supplement use. There were differences between the male and female population's behavior, the level of acceptance being higher in the case of the male population. This demonstrates the *third objective* of the study, namely: quantification of the effects felt by the consumers in relation to the destructuring of the biological processes after the consumption of these supplements.

The objectives of the study allow the delineation of the nutritional picture for people practicing physical activity, whose age is in the range of the young population for all levels of physical intensity, with an average height corresponding occasional practitioners on both the male and female segment (two peaks), and on the upper interval for frequently practitioners (three peaks).

The proposed model has, inherently, some limits related to the parameters selected in the questionnaire, which can be improved.

Authors aim at continuing the research to deepen the conclusions of the nutritional picture, increase the quality of nutrient supply, by highlighting the main characteristics that influence the consumption of these products.

## ACKNOWLEDGEMENTS

This work was supported by the project “Excellence and involvement in intelligent development, research and innovation at ‘Dunarea de Jos’ University of Galati”, “DINAMIC”, financed by the Romanian Ministry of Research and Innovation in the framework of Programme 1 – Development of the national research and development system, Sub‐programme 1.2 – Institutional Performance – Projects for financing excellence in Research, Development and Innovation, Contract no. 12PFE/2021.

## CONFLICT OF INTEREST

The authors declare that they have no known competing financial interests or personal relationships that could have appeared to influence the work reported in this article.

## Data Availability

The data that support the findings of this study are available from the corresponding author upon reasonable request.

## APPENDIX A

**Table:**

Question	Scale							
1. How often do you go to the gym?	a. Once a week	b. Once a month	c. More than four times a week		d. More than four times a month	e. I am an athlete		
2. What sport do you practice?	a. Fitness/strength/cardio	b. Aerobic (Tae‐Bo, Zumba, step aerobics, Kangoo jumps)	c. Spinning	d. X Body	e. Body pump	f. Bag boxing	g. Pilates	h. Other
3. What is the most important motivation for practicing your favorite sport?	a. The pleasure of practicing a sport	b. Aesthetic motivation		c. Medical motivation		d. Other? Which one		
4. Do you use nutritional supplements (sports)?	a. Yes			b. No				
5. You use nutritional supplements for:	a. Muscle building	b. Increased muscle strength	c. Weight loss		d. Recovery after effort	e. Decreasing the percentage of fat		
6. Do you think they will help you achieve your goal?	a. Yes, it gives me more energy	b. Yes, combined with diet helps me lose weight	c. Yes, combined with dieting it helps me build muscle		d. Yes, because I get the nutrients that I cannot assimilate by means of my daily diet	e. No		
7. Have you noticed any positive changes after the introduction of nutrient intake into your daily diet?	a. Yes, metabolic processes have accelerated by more than 10%,	b. Yes, metabolic processes have accelerated by more than 20%		c. Yes, metabolic processes have accelerated by more than 30%		d. No		
8. How often do you take supplements in the physical activity you do?	a. Weekly	b. Structured according to the intensity of the training program	c. I do not exceed the recommended daily dose		d. Daily	e. I replace the main meals with them		
9. Have you suffered any discomfort/side effects after taking dietary supplements? (more answers allowed)	a. Chest pain	b. Nausea	c. Vomiting	d. Cramps	e. Diarrhea	f. Body odor	g. No	
10. What types of dietary (sports) supplements do you take? (more answers allowed)	a. Proteins for muscle building	b. Proteins	c. Amino acids	d. Fat burning supplements	e. Sports performance supplements	f. Other. Please list other types of dietary (sports) supplements you use		
11. When choosing a nutritional supplement, the main motivation is:	a. Price accessibility	b. Recommendation of a specialist		c. I am loyal to a certain brand		d. I do not take nutritional supplements		
12. Where do you buy supplements from?	a. From pharmacies		b. From authorized distributors		c. From a friend			
13. Do you read the prospectus insert before taking dietary supplements?	a. Yes			b. No				
**Data about the respondent:**								
Age	.....years old							
Height				……cm				
Sex			F			M		
Marital status			Married			Single		
Occupation								
Education		High school		College		Tertiary	School	
Income		< 1000 lei		1000–2000	lei	<3000	lei	
